# Associations Between Diabetic Retinal Microvasculopathy and Neuronal Degeneration Assessed by Swept-Source OCT and OCT Angiography

**DOI:** 10.3389/fmed.2021.778283

**Published:** 2021-12-10

**Authors:** Bingjie Qiu, Lin Zhao, Xinyuan Zhang, Yanhong Wang, Qiyun Wang, Yao Nie, Xiaosi Chen, Carol Y. L. Cheung

**Affiliations:** ^1^Beijing Tongren Eye Center, Beijing Institute of Ophthalmology, Beijing Tongren Hospital, Capital Medical University, Beijing, China; ^2^ Beijing Retinal Choroidal Vascular Diseases Study Group; ^3^Department of Epidemiology and Biostatistics, Institute of Basic Medical Sciences Chinese Academy of Medical Sciences & School of Basic Medicine Peking Union Medical College, Beijing, China; ^4^Department of Ophthalmology and Visual Sciences, The Chinese University of Hong Kong, Hong Kong, China

**Keywords:** diabetic retinopathy, retinal neuronal degeneration, diabetic microvasculopathy, swept source optical coherence tomography, OCT angiography

## Abstract

**Purpose:** To provide clinical evidence of the associations between retinal neuronal degeneration and microvasculopathy in diabetic retinopathy (DR).

**Methods:** This case-control study included 76 patients (76 eyes) with type 2 diabetes mellitus (DM), and refraction error between −3.0 and +3.0 D. The eyes were assigned into DM (without DR), non-proliferative DR (NPDR), and proliferative DR (PDR) groups. Age-, sex-, and refractive error-matched normal subjects were enrolled as controls. The mean retinal thickness (mRT), the relative mean thickness of the retinal nerve fiber layer (rmtRNFL, mtRNFL/mRT), ganglion cell layer (rmtGCL), ganglion cell complex (rmtGCC) layer, foveal avascular zone area (FAZa), FAZ perimeter (FAZp), FAZ circularity index (FAZ-CI), and vessel density (VD) in superficial capillary plexus (SCP) and deep capillary plexus (DCP) were assessed by swept-source optical coherence tomography (OCT) and OCT angiography (OCTA). Group comparison and Spearman's partial correlation coefficient analysis were applied to evaluate the correlation between these morphological parameters.

**Results:** rmtRNFL, FAZa, and FAZp in SCP and DCP increased with the DR severity (*p*_*rmtRNFL*_ < 0.001; *p*_*FAZa, SCP*_ = 0.001; *p*_*FAZa*_, _*DCP*_ = 0.005; *p*_*FAZp*_, _*SCP*_ < 0.001; *p*_*FAZp*_, _*DCP*_ < 0.001). The rmtGCL, FAZ-CI in SCP and DCP, and VD in DCP decreased with the DR severity (*p*_*rmtGCL*_ = 0.002, *p*_*FAZ*−*CI*_, _*SCP*_ = 0.002; *p*_*FAZ*−*CI, DCP*_ < 0.001, *p*_*VD*_, _*DCP*_ < 0.001). After controlling age, sex, duration of diabetes, and hypertension, the rmtRNFL, FAZa in SCP and DCP, and FAZp in SCP and DCP were correlated with the severity of DR (*p* < 0.05), while VD in SCP and DCP, FAZ-CI, and rmtGCL were negatively correlated with the severity of DR (*p* < 0.05). The rmtGCL was negatively correlated with the FAZa in SCP (*r* = −0.34, *p* = 0.002) and DCP (*r* = −0.23, *p* = 0.033), and FAZp in SCP (*r* = −0.37, *p* = 0.001) and DCP (*r* = −0.32, *p* = 0.003), but positively correlated with VD in SCP (*r* = 0.26, *p* = 0.016), VD in DCP (*r* = 0.28, *p* = 0.012), and FAZ-CI in DCP (*r* = 0.31, *p* = 0.006).

**Conclusions:** rmtRNFL, FAZ-CI in SCP and DCP, and FAZp in SCP are strong predictors of the severity of DR. The ganglion cell body loss is highly correlated with increased FAZp and FAZa, decreased FAZ-CI, and reduced VD with the severity of DR.

## Introduction

Diabetic retinopathy (DR) is among the most significant and disabling chronic complications of diabetes mellitus (1, 2). Although cumulative evidence from basic science and clinical research suggested that DR is a neurovascular disease (3, 4), the correlation between diabetic retinal microvasculopathy and neuronal degeneration still remains uncertain, and their synergistic effect is yet to be illustrated in humans.

In previous studies, retinal neuronal degeneration and microvascular dysfunction have been shown to promote the occurrence and development of DR (5–8). We further confirmed that retinal ganglion cell is the most susceptible retinal neuronal cell type in response to hyperglycemia in a diabetic animal model (3). Clinical studies further confirmed that retinal neurons dysfunction occurred before retinal microvasculopathy using electroretinography (ERG) and visual field examinations (9–11). However, the underlying mechanisms and the correlation between diabetic retinal microvasculopathy and neuron degeneration are yet uncertain. In this study, advanced swept-source optical coherence tomography (SS-OCT) and OCT angiography (OCTA) were used to further investigate the correlation between the early and sensitive parameters of retinal microvasculopathy including the foveal avascular zone area (FAZa), FAZ perimeter (FAZp), FAZ circularity index (FAZ-CI), and retinal vessel density (VD) in superficial capillary plexus (SCP) and deep capillary plexus (DCP), and the parameters for evaluation of the early morphological changes of ganglion cells including the relative mean thickness of the retinal nerve fiber layer (rmtRNFL, mtRNFL/mRT), the relative mean thickness of the ganglion cell layer (rmtGCL), and the relative mean thickness of the ganglion cell complex layer (rmtGCC) in DR.

## Subjects and Methods

### Participants

This study followed the Ethical Principles for Medical Research Involving Human Subjects of the Declaration of Helsinki and was approved by the Ethics Committee of Beijing Tongren Hospital, Capital Medical University. All the enrolled subjects signed an informed consent form before participation.

A total of 76 patients (76 eyes) with type 2 diabetes mellitus (T2DM) were enrolled in this study, including 39 males and 37 females, aged 40–71 years (average 58.34 ± 7.47 years), with a refractive error between −3.0 diopter (D) and +3.0 D and registered in the outpatient clinic of Beijing Tongren Hospital from September 2018 to September 2019. Age-, sex-, and refractive error-matched 28 healthy subjects (28 eyes) [aged 43–78 years, median (interquartile, IQR) 53 (48–62) years] constituted the normal control group. If bilateral eyes were diagnosed as DR in patients with T2DM, only the severely affected eye was enrolled in the study.

### Sample Size Calculation

Combined with our pilot study results, which enrolled at least 15 subjects per group to show the statistical significance, Cochran's sample size formula was applied and the previous studies were referenced (27). Sample size was calculated at 95% confidence level with a margin of error of ±5%. We determined the minimum sample size to be 20 subjects in each group to detect the difference (0.83) between means of the OCT and OCTA parameters with a significance level of (alpha) 0.05.

### Inclusion and Exclusion Criteria

The inclusion criteria were as follows: (1) participants aged 40–70 years with T2DM; (2) refractive errors between −3.00 D and +3.00 D. T2DM was defined according to the American Diabetes Association (ADA) guidelines of DM (28–33), while DR was defined and classified by ophthalmologists according to the 2017 “Diabetic Retinopathy: A Position Statement of Diabetic Retinopathy” by the American Diabetes Association (34). Age-, sex-, and refractive error-matched normal subjects were enrolled as controls.

The exclusion criteria were as follows: (1) cysts detected in the neural retina by OCT; (2) macular edema secondary to any other retinal vascular diseases; (3) patients with other fundus diseases, such as age-related macular degeneration, uveitis, and retinal inherited diseases; (4) eyes with history of posterior surgery within 1 year; (5) eyes that underwent pan-retinal photocoagulation or focal/grid laser treatment; (6) eyes treated with intravitreal anti-vascular endothelial growth factor (VEGF) agent or steroid within one year; (7) patients with glaucoma or ocular hypertension; (8) fundus examination could not be performed due to ocular media opacities; (9) patients could not tolerate examinations due to severe systemic diseases.

### Eye Examination

#### Routine Eye Examinations

The best-corrected visual acuity (BCVA), non-contact intraocular pressure (TX20 Automatic Non-contact Tonometer, Canon Co., Ltd., Tokyo, Japan), slit-lamp microscopic examination (SL-IE Slit Lamp Microscope, Topcon Co., Ltd., Tokyo, Japan), and fundus examination with mydriasis and fundus photography (CR-1 non-mydriatic Fundus Camera, Canon Co., Ltd., Tokyo, Japan) were employed in all the enrolled subjects.

#### Detection and Quantification of the Morphological Parameters by OCT

**Figure 1:**
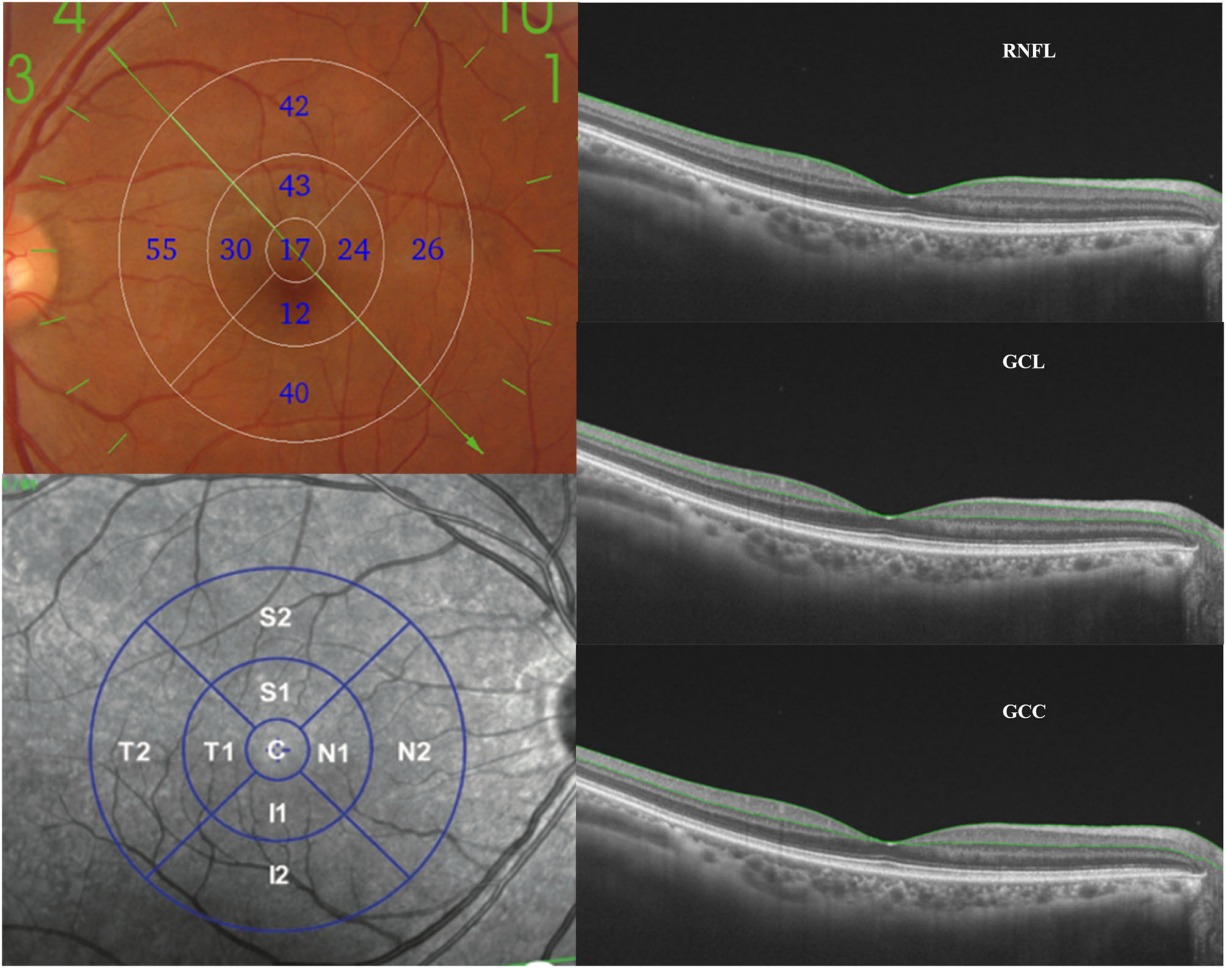
Measurement and analysis of the thickness of RNFL, GCL, and GCC layers. The ETDRS map (nine subfields) was utilized to calculate the average thickness of the RNFL, GCL, and GCC layers. RNFL, retinal nerve fiber layer; GCL, ganglion cell layer; GCC, ganglion cell complex layer; ETDRS, the Early Treatment Diabetic Retinopathy Study.

Imaging with DRI-Triton OCT: All subjects were examined by SS-OCT (DRI OCT1 Atlantis scanner, Topcon Co., Ltd., Tokyo, Japan). A 9 × 9 mm scanning range mode was selected to obtain B-scan images. The thickness of the retinal nerve fiber layer (RNFL), ganglion cell layer (GCL), and ganglion cell complex (GCC) in nine macular regions was measured automatically using TOPCON Advanced Boundary Segmentation (TABS) software. The nine regions were designed according to the Early Treatment Diabetic Retinopathy Study Classification (ETDRS) system. The ETDRS map was divided into nine parts: one 1 mm diameter circle at the center, outside four inner-quarter annuluses on the outside between the 1 and 3 mm diameter circles, and four outer-quarter annuluses between the 3 and 6 mm diameter circles (Figure 1). All images were measured independently by two ophthalmologists. Computer-aided manual correction of OCT segmentation was used for correcting thickness measurements in cases with errors.

#### Detection and Quantification of the OCTA Parameters

Optical coherence tomography angiography images were obtained using a 6 × 6 mm scanning mode in the macular area. Individual images in SCP, DCP, outer retina, and choriocapillaris plexus were generated by Topcon IMAGEnet^®^ 6 software with the computer-aided manual correction in cases with errors (35). FAZa and FAZp in SCP and DCP were measured by Image J software (version 1.48, available online at http://rsb.info.nih.gov/ij/, National Institutes of Health, Bethesda, MD, USA). Foveal avascular zone circularity index (FAZ-CI) (36) is an indicator and novel biomarker representing disruption of the parafoveal capillary network. The shape of FAZ is closer to a regular circular shape when FAZ-CI is close to 1. The formula of the FAZ-CI is:


$$\begin{array}{l} FAZ-CI=\frac{4\pi \;\times \;area}{perimeter^{2}} \end{array}$$

**Figure 2:**
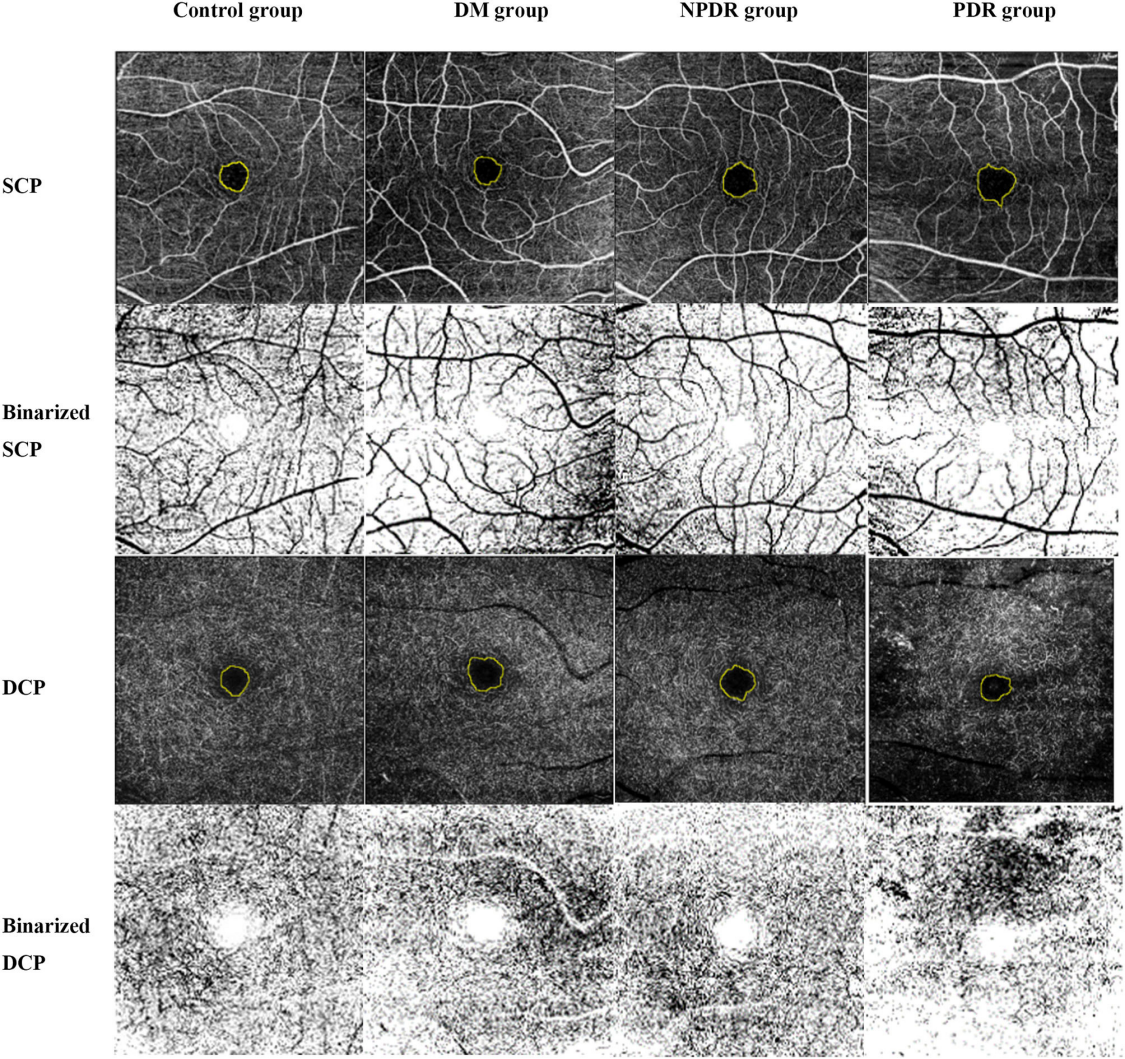
Representative OCTA images illustrate the quantification methods of VD, FAZa, FAZp, and FAZ-CI in SCP and DCP. OCTA images were obtained using a 6 × 6 mm scanning mode in the macular area. Individual images in SCP, DCP, outer retina, and choriocapillaris plexus were generated by Topcon IMAGEnet^®^ 6 software with the computer-aided manual correction in cases with errors in eyes with normal control (healthy subjects), DM (no DR), NPDR, and PDR. The FAZa and perimeter (yellow circle) were quantified by Image J software. Ostu method was applied to calculate the VD using Image J software. OCTA, optical coherence tomography angiography; VD, vessel density; FAZ, foveal avascular zone; FAZ-CI, FAZ circularity index; SCP, superficial capillary plexus; DCP, deep capillary plexus; DM, diabetes mellitus; DR, diabetic retinopathy; NPDR, non-proliferative diabetic retinopathy; PDR, proliferative diabetic retinopathy.

OCTA images were binarized with Ostu threshold method using Image J software (37). The VD in superficial and deep capillary networks was measured by calculating the proportion of vessel area with blood flow over the total area measured (38) (Figure 2).

To avoid individual differences and other confounding factors, we used the mean value (the average of ETDRS nine subfields) and relative thickness to describe the morphological parameters of retinal ganglion cells determined by SS-OCT. The relative mean thickness of RNFL (rmtRNFL), GCL (rmtGCL), and GCC (rmtGCC) was defined as the mean thickness of RNFL, GCL, or GCC/mean retinal thickness, respectively.

### Statistical Analysis

Statistical analysis was performed using SPSS software (SPSS, Inc. 24.0, Chicago, IL, USA). Age of patients, the duration of diabetes, the FAZa, rmtRNFL, rmtGCL, and rmtGCC in each group were described by means ± standard deviation (mean ± SD) or median (interquartile range). The comparisons between the groups were analyzed by one-way analysis of variance (ANOVA) or Kruskal–Wallis test. Spearman's partial correlation analysis was used to analyze the correlation between the parameters of retinal degeneration and microvasculopathy or between various morphological parameters with the severity of DR after controlling the age, gender, duration of diabetes, and hypertension. A value of *P* < 0.05 was considered statistically significant.

## Results

### Demographic Characteristics of the Enrolled Subjects

The DM group included 10 males (10 eyes) and 11 females (11 eyes) (aged 42–70 years, average: 59.62 ± 7.57 years), while 18 males (18 eyes) and 15 females (15 eyes) (aged 40–71 years, average: 59.76 ± 7.43 years) constituted the non-proliferative diabetic retinopathy (NPDR) group, and 11 males (11 eyes) and 11 females (11 eyes) (aged 43–70 years, average: 55.00 ± 6.63 years) constituted the proliferative diabetic retinopathy (PDR) group. In the control group, 28 patients (28 eyes) with normal age matching were selected, including 11 males (11 eyes) and 17 females (17 eyes) (aged 43–78 years, median (IQR) 53 (12–26) years) (Table 1).

**Table 1:** The demographic characteristics and the biochemical indicators of subjects.

	**DM**	**NPDR**	**PDR**	**Control**	***F/χ^2^***	***P* value**
	**(*N* = 21)**	**(*N* = 33)**	**(*N* = 22)**	**(*N* = 28)**		
Age, y [mean ± SD or median (interquartile range)]	59.62 ± 7.57	59.76 ± 7.43	55.00 ± 6.63	53 (48.62)	2.259^a^	0.086
Gender, female (*N*) (%)	11 (52.4)	15 (44.1)	11 (50.0)	17 (60.7)	1.720^c^	0.633
Duration of Hypertension≥15 years/Duration of Hypertension <15 years	6/10	14/18	5/16	–	2.197^c^	0.333
Duration of DM (mean ± SD)	11.58 ± 5.41	15.24 ± 7.51	14.32 ± 6.50	–	1.808^a^	0.171
Fasting blood glucose, mmol/L (mean ±SD)	8.10 ± 2.36	8.50 (6.35, 10.07)	8.06 ± 2.49	–	0.836^b^	0.658
HbA1c, % (mean ± SD)	8.02 ± 1.81	7.93 ± 1.25	8.46 ± 1.69	–	0.775^a^	0.464
RT, μm (mean ± SD)	282.17 ± 13.31	289.05 ± 20.54	293.43 ± 34.23	277.60 ± 14.56	2.663^a^	0.052

*Statistically significant: P ≤ 0.05. According to the type of data and the data distribution, ^a^one-way ANOVA analysis, Post-hoc Bonferroni's statistic, ^b^Kruskal–Wallis test, and ^c^Chi-square test were applied.

*SD, standard deviation; HbA1c, hemoglobin a1c; RT, retinal thickness; DM, diabetes mellitus; NPDR, non-proliferative diabetic retinopathy; PDR, proliferative diabetic retinopathy*.

No statistically significant difference was found in the gender and age among the four groups (*p*_*gender*_ = 0.633, *p*_*age*_ = 0.086). No significant difference was observed in the duration of DM in the DM, NPDR, and PDR groups (*p* = 0.171). Also, no significant difference was noted in the fasting blood glucose in the DM, NPDR, and PDR groups (*p* = 0.658). The glycosylated hemoglobin levels in the DM, NPDR, and PDR groups did not differ significantly (*p* = 0.464). Furthermore, no significant difference was detected in the duration of hypertension among the four groups (χ^2^ = 2.20, *p* = 0.333) (Table 1).

### Correlations of the Mean Macular Thickness With Diabetic Retinopathy Severity

According to the general trend, the mean macular thickness increased with DR severity. However, there was no statistically significant difference observed between the normal (277.60 ± 14.56 μm), DM (282.17 ± 13.31 μm), NPDR (289.05 ± 20.54 μm), and PDR (293.43 ± 34.23 μm) groups (277.60 ± 14.56 μm vs. 282.17 ± 13.31 μm vs. 289.05 ± 20.54 μm vs. 293.43 ± 34.23 μm, *p*_*all*_ = 0.052) (Table 1).

### Relative Mean RNFL Thickness Increased With Diabetic Retinopathy Severity

**Figure 3:**
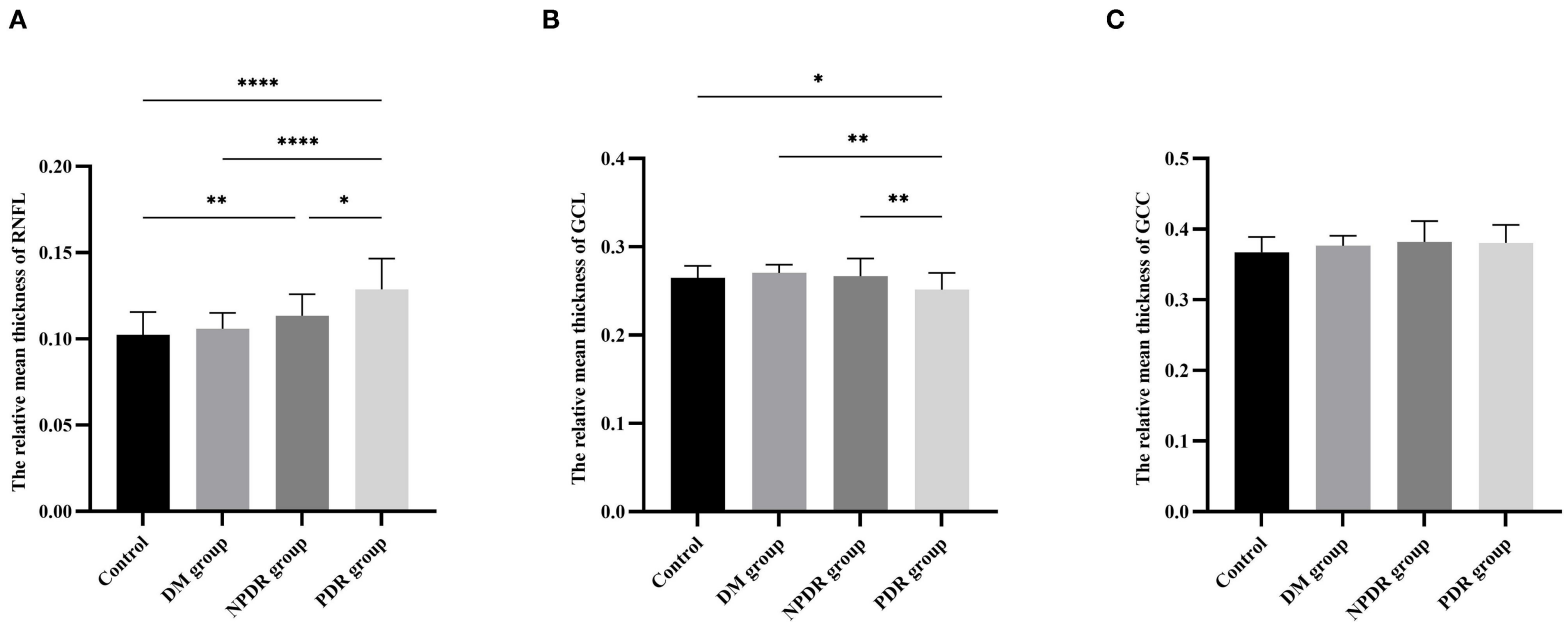
Comparisons of relative mean thickness of RNFL, GCL, and GCC layers among the DM, NPDR, PDR, and normal control groups. The bar charts show the comparison results of the relative mean thickness of RNFL **(A)**, GCL **(B)**, GCC **(C)** in eyes with normal control (healthy subjects), DM (no DR), NPDR, and PDR. RNFL, retinal nerve fiber layer; GCL, ganglion cell layer; GCC, ganglion cell complex layer; DM, diabetes mellitus; DR, diabetic retinopathy; NPDR, non-proliferative diabetic retinopathy; PDR, proliferative diabetic retinopathy. * *p* < 0.05, ** *p* < 0.01, *** *p* < 0.001.

The rmtRNFL thickness (ratio of the mean thickness of RNFL) in the DM group was 0.11 ± 0.01, and that in the NPDR, PDR, and normal control groups was 0.11 (0.11–0.12), 0.13 ± 0.02, and 0.10 (0.10–0.11), respectively. The rmtRNFL thickness in the normal group was significantly lower than that in the NPDR and PDR groups [0.11 (0.11-0.12) vs. 0.10 (0.10-0.11), *p*_*NPDRvs*.*Normal*_ = 0.007 and 0.13 ± 0.02 vs. 0.10 (0.10-0.11), *p*_*PDRvs*.*Normal*_ < 0.001]. The rmtRNFL thickness in the DM group was significantly lower than that in the PDR group [0.13 ± 0.02 vs. 0.11 ± 0.01, *p*_*PDRvs*.*DM*_ < 0.001]. These results indicated that the mean thickness of RNFL/RT increased gradually with an increase in severity of DR (Figure 3A, Table 2).

**Table 2:** Comparisons of the parameters of the ganglion cell degeneration and microvasculopathy in the normal (no DM), DM (no DR), NPDR, and PDR groups.

	**Control**	**DM**	**NPDR**	**PDR**	***F/χ2***	***P*-value**	
	**(*N = 28)***	**(*N* = 21)**	**(*N* = 33)**	**(*N* = 22)**	**(all)**	**(all)**	
rmtRNFL [mean ±SD or median (interquartile range)]	0.10 (0.10, 0.11)	0.11 ± 0.01	0.11 (0.11, 0.12)	0.13 ± 0.02	35.10^b^	<0.001^*^	
		*p _*DMvs*.*Normal*_*	*p_*NPDRvs*.*Normal*_*	*p_*PDRvs*.*Normal*_*	*p_*NPDRvs*.*DM*_*	*p_*PDRvs*.*DM*_*	*p_*PDRvs*.*NPDR*_*
		1.000	0.007*	**<0.001** ^*^	0.249	**<0.001** ^*^	**0.039** ^*^
rmtGCL [mean ±SD or median (interquartile range)]	0.26 ± 0.02	0.27 ± 0.01	0.27 ± 0.02	0.25 ± 0.02	5.38^a^	0.002^*^	
		*p _*DMvs*.*Normal*_*	*p_*NPDRvs*.*Normal*_*	*p_*PDRvs*.*Normal*_*	*p_*NPDRvs*.*DM*_*	*p_*PDRvs*.*DM*_*	*p_*PDRvs*.*NPDR*_*
		1.000	1.000	**0.040** ^*^	1.000	**0.002** ^*^	**0.009** ^*^
rmtGCC [mean ±SD or median (interquartile range)]	0.37 ± 0.02	0.38 ± 0.01	0.38 ± 0.03	0.38 ± 0.03	2.08^a^	0.108	
		*p _*DMvs*.*Normal*_*	*p_*NPDRvs*.*Normal*_*	*p_*PDRvs*.*Normal*_*	*p_*NPDRvs*.*DM*_*	*p_*PDRvs*.*DM*_*	*p_*PDRvs*.*NPDR*_*
		1.000	0.127	0.375	1.000	1.000	1.000
SCP vessel density, % (mean ± SD)	29.12 ± 3.98	27.88 ± 4.18	26.25 ± 4.94	26.25 ± 3.72	2.45^a^	0.067	
		*p _*DMvs*.*Normal*_*	*p_*NPDRvs*.*Normal*_*	*p_*PDRvs*.*Normal*_*	*p_*NPDRvs*.*DM*_*	*p_*PDRvs*.*DM*_*	*p_*PDRvs*.*NPDR*_*
		1.000	0.094	0.161	1.000	1.000	1.000
DCP vessel density, % [mean ± SD or median (interquartile range)]	30.77 ± 2.94	30.13 ± 2.81	28.58 ± 6.22	25.28 ± 2.76	24.846^b^	<0.001^*^	
		*p _*DMvs*.*Normal*_*	*p_*NPDRvs*.*Normal*_*	*p_*PDRvs*.*Normal*_*	*p_*NPDRvs*.*DM*_*	*p_*PDRvs*.*DM*_*	*p_*PDRvs*.*NPDR*_*
		1.000	0.144	<0.001*	0.457	**<0.001** ^*^	**0.050** ^*^
FAZ area in superficial capillary plexus, mm^2^ [mean ± SD or median (interquartile range)]	0.36 ± 0.14	0.34 ± 0.10	0.47 ± 0.17	0.57 ± 0.25	17.54^b^	0.001^*^	
		*p _*DMvs*.*Normal*_*	*p_*NPDRvs*.*Normal*_*	*p_*PDRvs*.*Normal*_*	*p_*NPDRvs*.*DM*_*	*p_*PDRvs*.*DM*_*	*p_*PDRvs*.*NPDR*_*
		1.000	0.095	**0.002** ^*^	0.197	**0.006** ^*^	0.846
FAZ area in deep capillary plexus, mm^2^ (mean ± SD or median (interquartile range)]	0.52 ± 0.21	0.59 ± 0.16	0.69 ± 0.21	0.79 ± 0.31	12.83^b^	0.005^*^	
		*p _*DMvs*.*Normal*_*	*p_*NPDRvs*.*Normal*_*	*p_*PDRvs*.*Normal*_*	*p_*NPDRvs*.*DM*_*	*p_*PDRvs*.*DM*_*	*p_*PDRvs*.*NPDR*_*
		1.000	**0.040** ^*^	**0.007** ^*^	0.873	0.227	1.000
FAZ perimeter in superficial capillary plexus, mm [mean ± SD or median (interquartile range)]	2.29 ± 0.49	2.32 ± 0.33	2.86 ± 0.54	3.15 (2.67, 4.07)	23.82^b^	<0.001^*^	
		*p _*DMvs*.*Normal*_*	*p_*NPDRvs*.*Normal*_*	*p_*PDRvs*.*Normal*_*	*p_*NPDRvs*.*DM*_*	*p_*PDRvs*.*DM*_*	*p_*PDRvs*.*NPDR*_*
		1.000	**0.005** ^*^	**<0.001** ^*^	0.057	**0.004** ^*^	1.000
FAZ perimeter in deep capillary plexus, mm [mean ± SD or median (interquartile range)]	2.67 ± 0.61	3.19 (2.80, 3.31)	3.31 (2.81, 3.94)	4.14 ± 1.34	22.92^b^	<0.001^*^	
		*p _*DMvs*.*Normal*_*	*p_*NPDRvs*.*Normal*_*	*p_*PDRvs*.*Normal*_*	*p_*NPDRvs*.*DM*_*	*p_*PDRvs*.*DM*_*	*p_*PDRvs*.*NPDR*_*
		0.557	**0.009** ^*^	**<0.001** ^*^	1.000	**0.023** ^*^	0.392
FAZ-CI in superficial capillary plexus [mean ± SD or median (interquartile range)]	0.82 ± 0.10	0.79 ± 0.08	0.71 ± 0.13	0.65 ± 0.21	14.50^b^	0.002^*^	
		*p _*DMvs*.*Normal*_*	*p_*NPDRvs*.*Normal*_*	*p_*PDRvs*.*Normal*_*	*p_*NPDRvs*.*DM*_*	*p_*PDRvs*.*DM*_*	*p_*PDRvs*.*NPDR*_*
		1.000	**0.011** ^*^	**0.008** ^*^	0.395	0.241	1.000
FAZ-CI in deep capillary plexus [mean ± SD or median (interquartile range)]	0.88 ± 0.04	0.78 ± 0.08	0.75 ± 0.10	0.63 ± 0.19	36.54^b^	<0.001^*^	
		*p _*DMvs*.*Normal*_*	*p_*NPDRvs*.*Normal*_*	*p_*PDRvs*.*Normal*_*	*p_*NPDRvs*.*DM*_*	*p_*PDRvs*.*DM*_*	*p_*PDRvs*.*NPDR*_*
		**0.004** ^*^	**<0.001** ^*^	**<0.001** ^*^	1.000	0.090	0.336

* *Statistically significant: P ≤ 0.05. According to the type of data and the data distribution, ^a^one-way ANOVA analysis, Post-hoc Bonferroni's statistic, ^b^Kruskal–Wallis test, and ^c^Chi-square test were applied*.

*SD, standard deviation; rmtRNFL, relative mean thickness of retinal nerve fiber layer; rmtGCL, relative mean thickness of ganglion cell layer; rmtGCC, relative mean thickness of ganglion cell complex; SCP, superficial capillary plexus; DCP, deep capillary plexus; FAZ, foveal avascular zone; FAZ-CI, circularity index of the foveal avascular zone; DM, diabetes mellitus; NPDR, non-proliferative diabetic retinopathy; PDR, proliferative diabetic retinopathy*.

### Relative Mean GCL Thickness Decreased With Diabetic Retinopathy Severity

The rmtGCL thickness (GCL/RT) layer was 0.27 ± 0.01 in the DM group, 0.27 ± 0.02 in the NPDR group, 0.25 ± 0.02 in the PDR group, and 0.26 ± 0.02 in the normal control group, respectively. The rmtGCL thickness was significantly lower in the PDR group than that in the normal control, DM, and NPDR groups (0.25 ± 0.02 vs. 0.26 ± 0.02, *p*_*PDRvs*.*Normal*_ = 0.040; 0.25 ± 0.02 vs. 0.27 ± 0.01, *p*_*PDRvs*.*DM*_ = 0.002; 0.25 ± 0.02 vs. 0.27 ± 0.02, *p*_*PDRvs*.*NPDR*_ = 0.009, respectively) (Figure 3B, Table 2).

### Correlations of the Mean GCC Thickness With Diabetic Retinopathy Severity

No statistically significant difference was observed in the rmtGCC thickness in the normal control (0.37 ± 0.02), DM (0.38 ± 0.01), NPDR (0.38 ± 0.03), and PDR (0.38 ± 0.03) groups (0.37 ± 0.02 vs. 0.38 ± 0.01 vs. 0.38 ± 0.03 vs. 0.38 ± 0.03, *p* = 0.108) (Figure 3C, Table 2).

### Vessel Density in SCP and DCP Decreased With Diabetic Retinopathy Severity

**Figure 4:**
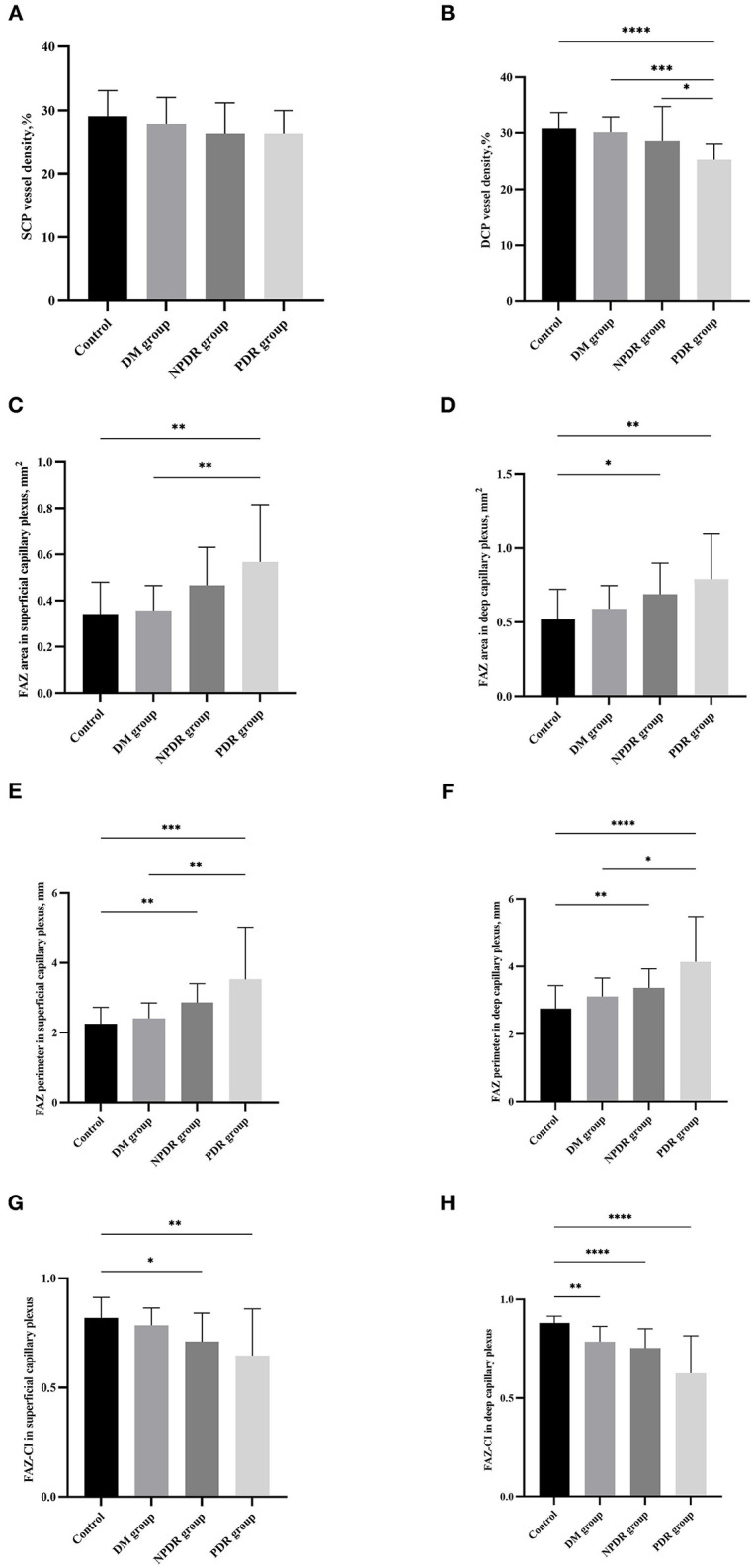
Comparisons of the retinal microvasculopathy (FAZa, FAZp, FAZ-CI, and VD in SCP and DCP) among the normal control (healthy subjects), DM (no DR), NPDR and PDR groups. The bar charts show the comparison results of the VD in SCP **(A)**, VD in DCP **(B)**, FAZa in SCP **(C)**, FAZa in DCP **(D)**, FAZp in SCP **(E)**, FAZp in DCP **(F)**, FAZ-CI in SCP **(G)**, and FAZ-CI in DCP **(H)** in eyes with normal control (healthy subjects), DM (no DR), NPDR, and PDR. FAZ, foveal avascular zone; FAZ-CI, FAZ circularity index; VD, vessel density; SCP, superficial capillary plexus; DCP, deep capillary plexus; DM, diabetes mellitus; DR, diabetic retinopathy; NPDR, non-proliferative diabetic retinopathy; PDR, proliferative diabetic retinopathy.

Superficial capillary plexus vessel density was 29.12 ± 3.98% in the normal control, 27.88 ± 4.18% in DM, 26.25 ± 4.94% in NPDR, and 26.25 ± 3.72% in PDR, respectively. From these results, we found that the SCP vessel density decreased with diabetic retinopathy severity while there was no significant difference between the four groups (29.12 ± 3.98% vs. 27.88 ± 4.18% vs. 26.25 ± 4.94% vs. 26.25 ± 3.72%, *p*_*all*_ = 0.067) (Figure 4A, Table 2).

Deep capillary plexus vessel density was 30.77 ± 2.94% in the normal control, 30.13 ± 2.81% in DM, 28.58 ± 6.22% in NPDR, and 25.28 ± 2.76% in PDR, respectively. A significant difference was observed between the four groups (30.77 ± 2.94% vs. 30.13 ± 2.81% vs. 28.58 ± 6.22% vs. 25.28 ± 2.76%, *p*_*all*_ < 0.001). DCP vessel density is significantly higher than that in the PDR groups (25.28 ± 2.76% vs. 30.77 ± 2.94%, *p*_*PDRvs*.*Normal*_ < 0.001). DCP vessel density in the DM group was significantly larger than that in PDR group (25.28 ± 2.76% vs. 30.13 ± 2.81%, *p*_*PDRvs*.*DM*_ < 0.001), and the value in the NPDR group was significantly larger than that in the PDR group (25.28 ± 2.76% vs. 28.58 ± 6.22%, *p*_*PDRvs*.*NPDR*_ = 0.050) (Figure 4B, Table 2).

### Correlations of FAZ Area in SCP and DCP With Diabetic Retinopathy Severity

The value of FAZa in SCP was 0.36 ± 0.14 mm^2^ in the normal control, 0.34 ± 0.10 mm^2^ in DM, 0.47 ± 0.17 mm^2^ in NPDR, and 0.57 ± 0.25 mm^2^ in PDR, respectively. A significant difference was observed between the four groups (0.36 ± 0.14 mm^2^ vs. 0.34 ± 0.10 mm^2^ vs. 0.47 ± 0.17 mm^2^ vs. 0.57 ± 0.25 mm^2^, *p*_*all*_ = 0.001). The FAZa in the PDR group was significantly enlarged than that in the DM (0.57 ± 0.25 mm^2^ vs. 0.34 ± 0.10 mm^2^, *p*_*PDRvs*.*DM*_ = 0.006) and normal control (0.57 ± 0.25 mm^2^ vs. 0.36 ± 0.14 mm^2^, *p*_*PDRvs*.*Normal*_ = 0.002) groups (Figure 4C, Table 2).

The value of FAZa in DCP was 0.52 ± 0.21 mm^2^ in the normal control, 0.59 ± 0.16 mm^2^ in DM, 0.69 ± 0.21 mm^2^ in NPDR, and 0.79 ± 0.31 mm^2^ in PDR, respectively. There was a significant difference between the four groups (0.52 ± 0.21 mm^2^ vs. 0.59 ± 0.16 mm^2^ vs. 0.69 ± 0.21 mm^2^ vs. 0.79 ± 0.31 mm^2^, *p*_*all*_ = 0.005). The FAZa in DCP in the normal control group was significantly smaller than that in the NPDR (0.69 ± 0.21 mm^2^ vs. 0.52 ± 0.21 mm^2^, *p*_*NPDRvs*.*Normal*_ = 0.040) and PDR (0.79 ± 0.31 mm^2^ vs. 0.52 ± 0.21 mm^2^, *p*_*PDRvs*.*Normal*_ = 0.007) groups (Figure 4D, Table 2).

### Correlations of FAZ Perimeter in SCP and DCP With Diabetic Retinopathy Severity

The FAZp in SCP was 2.29 ± 0.49 mm in the normal control group, 2.32 ± 0.33 mm in DM, 2.86 ± 0.54 mm in NPDR, and 3.15 (2.67–4.07) mm in PDR, respectively. A significant difference was detected between the four groups [2.29 ± 0.49 mm vs. 2.32 ± 0.33 mm vs. 2.86 ± 0.54 mm vs. 3.15 (2.67–4.07) mm, *p*_*all*_ < 0.001]. The FAZp in SCP layer in the NPDR and PDR groups were significantly enlarged than that in the normal group [2.86 ± 0.54 mm vs. 2.29 ± 0.49 mm, *p*_*NPDRvs*.*Normal*_ = 0.005 and 3.15 (2.67–4.07) mm vs. 2.29 ± 0.49 mm, *p*_*PDRvs*.*Normal*_ < 0.001, respectively]. The FAZp in SCP layer in the PDR group was significantly enlarged than that in the DM group [3.15 (2.67–4.07) mm vs. 2.32 ± 0.33 mm, *p*_*PDRvs*.*DM*_ = 0.004] (Figure 4E, Table 2).

The value of FAZp in DCP was 2.67 ± 0.61 mm in the normal control, 3.19 (2.80–3.31) mm in DM, 3.31(2.81–3.94) mm in NPDR, and 4.14 ± 1.34 mm in PDR, respectively. There was a significant difference between the four groups (2.67 ± 0.61 mm vs. 3.19 (2.80–3.31) mm vs. 3.31 (2.81–3.94) mm vs. 4.14 ± 1.34 mm, *p*_*all*_ < 0.001). The FAZp in DCP in the NPDR and PDR group was significantly enlarged than that in the normal group [3.31(2.81–3.94) mm vs. 2.67 ± 0.61 mm, *p*_*NPDRvs*.*Normal*_ = 0.009 and 4.14 ± 1.34 mm vs. 2.67 ± 0.61 mm, *p*_*PDRvs*.*Normal*_ < 0.001, respectively]. The FAZp in DCP in the PDR group was significantly enlarged than that in the DM group [4.14 ± 1.34 mm vs. 3.19 (2.80–3.31) mm, *p*_*PDRvs*.*DM*_ = 0.023] (Figure 4F, Table 2).

### FAZ-CI in SCP and DCP Decreased With Diabetic Retinopathy Severity

The value of FAZ-CI in SCP was 0.82 ± 0.10 in the normal control, 0.79 ± 0.08 in DM, 0.71 ± 0.13 in NPDR, and 0.65 ± 0.21 in PDR, respectively. A significant difference was observed between the four groups (0.82 ± 0.10 vs. 0.79 ± 0.08 vs. 0.71 ± 0.13 vs. 0.65 ± 0.21, *p*_*all*_ = 0.002). The FAZ-CI in SCP in the normal control group was significantly bigger than that in the NPDR (0.71 ± 0.13 vs. 0.82 ± 0.10, *p*_*NPDRvs*.*Normal*_ = 0.011) and PDR (0.65 ± 0.21 vs. 0.82 ± 0.10, *p*_*PDRvs*.*Normal*_ = 0.008) groups (Figure 4G, Table 2).

The value of FAZ-CI in DCP was 0.88 ± 0.04 in the normal control, 0.78 ± 0.08 in DM, 0.75 ± 0.10 in NPDR, and 0.63 ± 0.19 in PDR, respectively. A significant difference was observed between the four groups (0.88 ± 0.04 vs. 0.78 ± 0.08 vs. 0.75 ± 0.10 vs. 0.63 ± 0.19, *p*_*all*_ < 0.001). The FAZ-CI in DCP in the normal control group was significantly bigger than that in the DM (0.78 ± 0.08 vs. 0.88 ± 0.04, *p*_*DMvs*.*Normal*_ = 0.004), NPDR (0.75 ± 0.10 vs. 0.88 ± 0.04, *p*_*NPDRvs*.*Normal*_ < 0.001), and PDR (0.63 ± 0.19 vs. 0.88 ± 0.04, *p*_*PDRvs*.*Normal*_ < 0.001) groups (Figure 4H, Table 2).

### Correlation Between Microvasculopathy and Neuronal Degeneration Among Different Groups

After controlling the age, sex, duration of diabetes, and hypertension, Spearman's partial coefficient correlation analysis showed that the rmtRNFL (*r* = 0.52, *p* < 0.001); FAZa in SCP (*r* = 0.34, *p* < 0.001) and in DCP (*r* = 0.26, *p* = 0.015); and FAZp in SCP(*r* = 0.43, *p* < 0.001) and in DCP (*r* = 0.36, *p* = 0.001) were positively significantly correlated with the severity of DR. SCP vessel density (*r* = −0.24, *p* = 0.027), DCP vessel density (*r* = −0.39, *p* < 0.001), FAZ-CI in SCP(*r* = −0.44, *p* < 0.001), DCP (*r* = −0.46, *p* < 0.001), and rmtGCL (*r* = −0.24, *p* = 0.022) were negatively correlated with the severity of DR.

**Figure 5:**
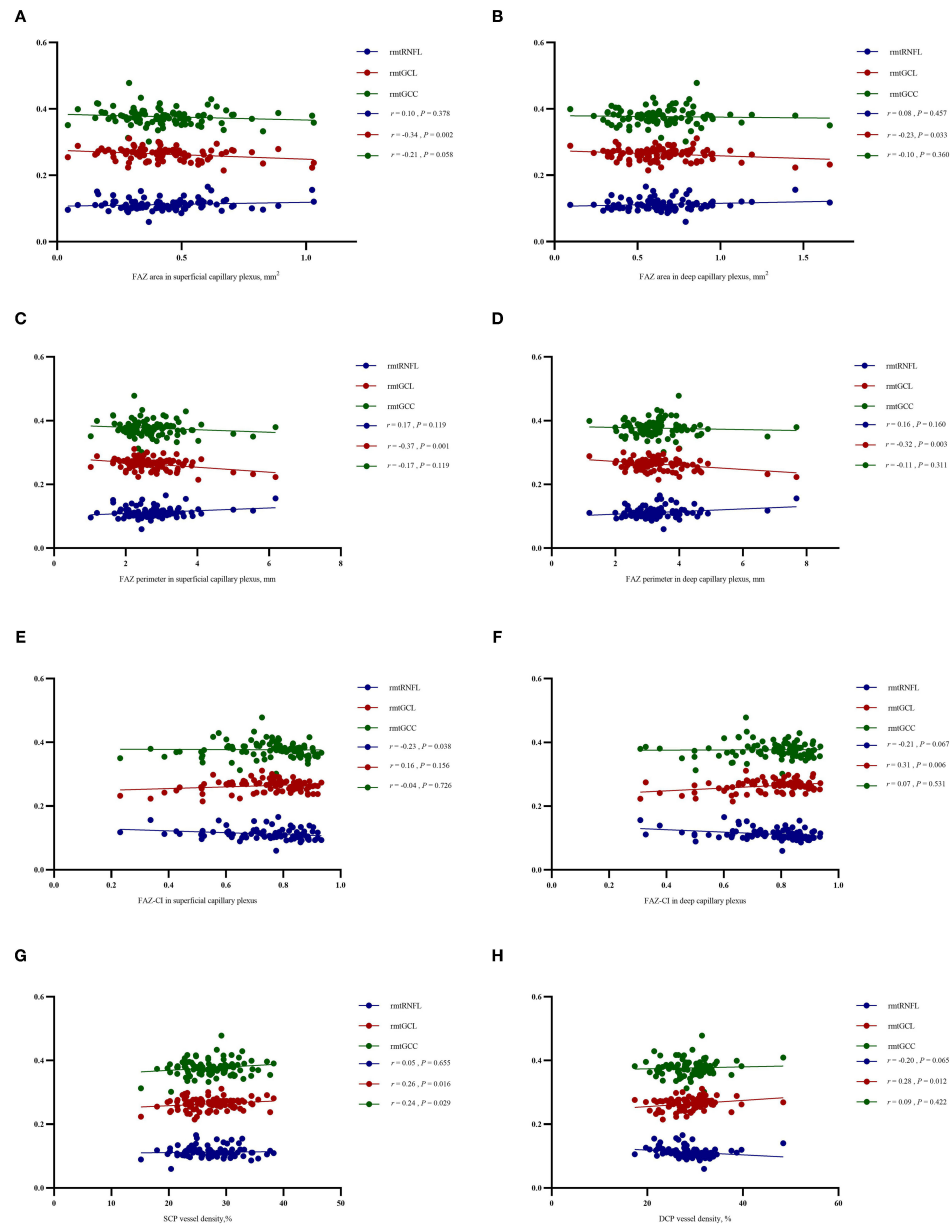
Relationship between the parameters of retinal microvasculopathy (FAZa, FAZp, FAZ-CI, and VD in SCP and DCP) and ganglion cell degeneration (the relative mean thickness of RNFL, GCL, and GCC). After controlling the age, sex, and duration of DM and hypertension with Spearman's partial coefficient correlation analysis, the rmtGCL was negatively correlated with the FAZa in SCP (*r* = −0.34, *P* = 0.002) and DCP (*r* = −0.23, *P* = 0.033), and FAZp in SCP (*r* = −0.37, *P* = 0.001) and DCP (*r* = −0.32, *P* = 0.003), but positively correlated with VD in SCP (*r* = 0.26, *P* = 0.016) and DCP (*r* = 0.28, *P* =0.012), and FAZ-CI in DCP (*r* = 0.31, *P* = 0.006). The correlations between rmtRNFL, rmtGCL and rmtGCC with the FAZa in SCP **(A)**, FAZa in DCP **(B)**, FAZp in SCP **(C)**, FAZp in DCP **(D)**, FAZ-CI in SCP **(E)**, FAZ-CI in DCP **(F)**, VD in SCP **(G)** and VD in DCP **(H)** were described. The rmtRNFL was negatively correlated with FAZ-CI in SCP (*r* = −0.23, *P* = 0.038). The rmtGCC was positively correlated with SCP vessel density (*r* = 0.24, *P* = 0.029). FAZ, foveal avascular zone; FAZ-CI, FAZ circularity index; VD, vessel density; SCP, superficial capillary plexus; DCP, deep capillary plexus; rmtRNFL, relative mean thickness of retinal nerve fiber layer; rmtGCL, relative mean thickness of ganglion cell layer; rmtGCC, relative mean thickness of ganglion cell complex; DM, diabetes mellitus.

The rmtGCL was significantly negatively correlated with the FAZa in SCP (*r* = −0.34, *p* = 0.002) and DCP (*r* = −0.23, *p* = 0.033), and with FAZp in SCP (*r* = −0.37, *p* = 0.001) and DCP (*r* = −0.32, *p* = 0.003). However, the rmtGCL was positively correlated with SCP vessel density (*r* = 0.26, *p* = 0.016) and DCP vessel density (*r* = 0.28, *p* = 0.012), and FAZ-CI in DCP (*r* = 0.31, *p* = 0.006) (Figure 5).

## Discussion

In this study, we found that increased rmtRNFL, FAZa, and FAZp, decreased rmtGCL, VD, and FAZ-CI are correlated with the severity of DR. rmtRNFL, FAZ-CI in SCP and DCP, and FAZp in SCP are stronger predictors of the severity of DR than the FAZa. The ganglion cell body loss (rmtGCL) is highly correlated with increased FAZa and perimeter, decreased FAZ-CI, and reduced VD with the severity of DR. These results indicated that diabetic microvasculopathy is highly correlated with neuronal degeneration, and neuron protection is an essential strategy to improve prevention and management in DR (3).

Neurovascular unit (NVU) is a new concept in neuroscience introduced by the Stroke Progress Review Group (39). The NVU is a complex structural and functional unit that regulates regional cerebral blood flow (CBF) and the delivery of nutrients (40). The dysfunction of NVU has been implicated in several diseases, such as stroke and Alzheimer's disease (41). Based on the concept of brain NVU and the close correlation between the retina and the brain in all aspects of function and anatomy, the concept of retinal neuronal vascular unit (RNVU) was proposed (42, 43). Neurons and endothelial cells are the critical components of RNVU. The interactions between neurons and microvasculature in DR have gained increasing attention in recent years. In this study, we found that the ganglion cell body loss is highly correlated with the retinal vasculature changes (increased FAZp, decreased FAZ-CI, and reduced VD) with the severity of DR, which is consistent with previous reports (44, 45).

A ganglion cell is mainly composed of a cell body (soma), a single axon, and dendrites. The electrical impulses transmitted by neurons mainly depend on axon transport, the process by which nerve cells transfer substances from the cell body to the axon tip. This ATP-dependent process plays an important role in maintaining the physiological activities of neurons and can occur in two delivery directions: anterograde (from the cell body to the axon tips) and retrograde (from the axon tips back to the cell body) transport. The microtubule cytoskeleton and motor proteins are critical for long-range axon transport.

The axons of the GCL constitute RNFL. Increasing evidence speculated that mitochondrial dysfunction and axoplasmic flow stasis in optic neuropathy (44, 45) lead to axonal swelling and contribute to most of the neurodegenerative diseases including DR (46–49). Eyes with early Leber Hereditary Optic Neuropathy (E-LHON) showed a thicker RNFL, but a thinner RNFL was seen in eyes with atrophic LHON (A-LHON) in comparison with the control group, thereby suggesting that the changes in RNFL thickness represent the different course of the disease (50). In a diabetic rat model, expression level of the synaptic proteins including vesicular glutamate transporter-1, syntaxin-1, and synaptotagmin-1 was decreased in the GCL, but the level of dynein motor proteins increased in the GCL after 8 weeks of diabetes, indicating that axonal transport contributes to early signs of neural dysfunction in the diabetic rat retinas (51). Furthermore, the degree of increased thickness of RNFL is closely correlated with visual evoked potential (VEP) amplitude loss and degree of optic atrophy, indicating that during the progression of DR, a thinner RNFL is a sign of optic atrophy following the swelling stage of axons of ganglion cells (52), and the degree of thickened RNFL may be an indicator of optic atrophy for patients with DR. Furthermore, in a primate model of human non-arteritic anterior ischemic optic neuropathy, RNFL swelling is confirmed as a strong and independent predictor of optic nerve atrophy, which agrees with our results (52). In this study, we have shown that the relative thickness of RNFL increased with DR severity concomitant with the decreased thickness of the GCL layer. This phenomenon suggested that the swelling of the GCL axon preceded the GCL cell body loss under hyperglycemia, thereby reflecting the unique hypoglycemia-mediated pathological damage of GCL. The pathological evidence, either in animal models or humans, is consistent with the findings of the current study (53–57).

Furthermore, FAZp, FAZ-CI, and VD are good sensors of retinal ischemia and have been implicated in several clinical studies to predict disease progression (58–60). Increased FAZp, FAZ-CI, and VD have been found to be an earlier sign of diabetic retinal vasculopathy (60–62) and are highly correlated with the severity of DR (12, 62). In comparison with FAZa, which significantly varies in the population, FAZ-CI is a better measurement index to assess FAZ circularity with a value range from 0 to 1 (13). FAZ-CI has been found to be tightly correlated with visual acuity in patients with DR (13, 14). We also found that enlargement of FAZp in SCP and DCP was correlated with DR severity as well as the duration of diabetes, which is in parallel to the previously published data (15–17). In previous studies, SCP and DCP vessel density was demonstrated to be decreased in DM (18) and highly correlated with DR progression (19), which are consistent with our results. We further demonstrated that microvascular injury is associated with GCL cell body loss in DR, providing the evidence for the first time that there is an interaction between retinal microvasculopathy and neuronal degeneration. Interestingly, we also found that in comparison with RNGL, GCL has a closer relationship with the vasculature ischemic parameters (FAZp, FAZ-CI, VD), indicating that the ganglion cell body is a good sensor for retinal ischemia.

The rapid revolution of OCT and OCTA has provided a powerful weapon for evaluating the morphological and structural changes in the various layers of the retina (20–22). Compared to traditional OCT, SS-OCT provides longer and deeper detection ability, ultra-high image acquisition speed, and a more powerful function of eliminating motion artifacts, thereby providing a distinct retinal microstructure (20, 23–26). OCTA has the advantage of investigating retinal vasculature non-invasively layer-by-layer (Figure 2). Automatic quantitative OCTA metrics have provided valuable information for disease progression beyond that could be obtained from fundus fluorescein angiography (FFA) (63).

In this study, to avoid the individual differences, the inter-eye correlation, and the other confounding factors, we used the following: (1) average value of ETDRS nine subfields to describe the thickness of the retina, RNFL, GCL, and GCC (64, 65); (2) all the values were automatically provided by Topton triton SS-OCT TABS software; (3) the relative mean thickness of RNFL, GCL, and GCC was defined as the mean thickness of RNFL, GCL, or GCC/mean retinal thickness. Gabriele et al. described a relative method in which RNFL thickness profiles relative to the distance from the disc center were computed for quadrants and clock hours (66). In our pilot study, a linear trend was established among the four groups, which was similar between the relative mean ETDRS macular thickness and absolute thickness; however, data distribution was based on relative thickness and indicated the differences among the groups.

Although the current study successfully showed the correlation between diabetic retinal vasculopathy and neuronal degeneration, these findings are limited due to the case-control design, and the causal relationship between neuronal degeneration and microvasculopathy cannot be determined, hence future validation using longitudinal data is warranted. Furthermore, the small sample size could not eliminate some confounding factors in the analysis, and so well-designed and large-scale prospective clinical trials are also needed to support the conclusions in the study. Morphological (OCT/OCTA) and functional evaluations (ERG) of neurons in a cohort study could also be considered to further determine the pathological changes in other neuronal cell types under hyperglycemia.

In summary, we found that there was a close correlation between diabetic retinal microvasculopathy and neuronal degeneration with respect to the occurrence and severity of DR in humans. The current results also hinted that neuroprotection is also critical for the prevention and managing of DR. Further, well-designed, large cohort studies are warranted to validate the findings of this study.

## Data Availability Statement

The original contributions presented in the study are included in the article/supplementary materials, further inquiries can be directed to the corresponding author/s.

## Ethics Statement

The studies involving human participants were reviewed and approved by Beijing Tongren Hospital, Capital Medical University. The patients/participants provided their written informed consent to participate in this study.

## Author Contributions

XZ contributed to the conception and design of the study and revision of the manuscript. BQ and LZ organized the database and drafted the manuscript. YW and BQ performed the statistical analysis. QW, YN, XC, and CC helped to enroll subjects. All authors contributed to manuscript revision, and read approved the submitted version.

## Funding

This work was supported by the National Natural Science Foundation of China [Grants 81570850, 81170859, and 82070988] and the Ministry of Science and Technology Foundation of China [Grant 2016YFC1305604].

## Conflict of Interest

The authors declare that the research was conducted in the absence of any commercial or financial relationships that could be construed as a potential conflict of interest.

## Publisher's Note

All claims expressed in this article are solely those of the authors and do not necessarily represent those of their affiliated organizations, or those of the publisher, the editors and the reviewers. Any product that may be evaluated in this article, or claim that may be made by its manufacturer, is not guaranteed or endorsed by the publisher.

## Abbreviations

ADA: American Diabetes Association
ANOVA: One-way analysis of variance
BCVA: Best-corrected visual acuity
CBF: Cerebral blood flow
D: Diopter
DCP: Deep capillary plexus
DM: Diabetes mellitus
DR: Diabetic retinopathy
ERG: Electroretinography
ETDRS: Early Treatment Diabetic Retinopathy Study Classification
FAZ: Foveal avascular zone
FAZa: Foveal avascular zone area
FAZ-CI: Foveal avascular zone-circularity index
FAZp: Foveal avascular zone perimeter
FFA: Fundus fluorescein angiography
GCC: Ganglion cell complex
GCL: Ganglion cell
ICP: Intracranial pressure
IIH: Idiopathic intracranial hypertension
IQR: Interquartile
mRT: Mean retinal thickness
NPDR: Non-proliferative diabetic retinopathy
NVU: Neurovascular unit
OCT: Optical coherence tomography
OCTA: Optical coherence tomography angiography
PDR: Proliferative diabetic retinopathy
rmtGCC: Relative mean thickness of the ganglion cell complex
rmtGCL: Relative mean thickness of the ganglion cell
rmtRNFL: Relative mean thickness of the retinal nerve fiber
RNFL: Retinal nerve fiber
RNVU: Retinal neuronal vascular unit
SCP: Superficial capillary plexus
SS-OCT: Swept-source optical coherence tomography
T2DM: Type 2 diabetes mellitus
TABS: TOPCON Advanced Boundary Segmentation
VD: Vessel density
VEP: Visual evoked potential.
